# Topical rifampicin for prevention of deep sternal wound infections in patients undergoing coronary artery bypass grafting

**DOI:** 10.1038/s41598-020-64310-y

**Published:** 2020-05-04

**Authors:** Luca Salvatore De Santo, Antonino Salvatore Rubino, Michele Torella, Marisa De Feo, Viviana Galgano, Nicola Guarente, Emilio Mango, Leonardo Savarese, Francesco Iorio, Carlo Zebele

**Affiliations:** 1 University of Campania Luigi Vanvitelli, Department of Translational Medical Sciences, V Monaldi Hospital, Cardiac Surgery Unit, Naples, Italy; 2 Casa di Cura Montevergine, Intensive Care Unit, Mercogliano, Avellino, Italy; 3 Clinica Montevergine, Cardiac Surgery Unit, Mercogliano, Avellino, Italy

**Keywords:** Cardiology, Medical research

## Abstract

Deep sternal wound infections (DSWI), although an infrequent complication, significantly impair postoperative outcomes after coronary artery bypass grafting (CABG) surgery. Among several preventive strategies, topical antibiotic therapy immediately before sternal closure has been strongly advocated. In this retrospective analysis, the incidence of DSWI in 517 patients undergoing isolated CABG and receiving rifampicin irrigation of mediastinum, sternum and suprasternal tissues was compared to an historical consecutive cohort of 448 patients. To account for the inherent selection bias, a 1:1 propensity matched analysis was performed. Patients receiving topical rifampicin experienced significantly less occurrence of postoperative DSWI (0.2% vs 2.5%, p = 0.0016 in the unmatched analysis; 0.3% vs 2.1%, p = 0.0391 in the matched analysis). Intensive care unit stay, hospital stay, and operative mortality were similar between groups. This study shows that topical rifampicin in combination with commonly prescribed preventative strategies significantly reduces the incidence of DSWI to less than 0.3% in unselected patients undergoing a full median sternotomy for CABG. Further studies, including a larger number of patients and with a randomization design, would establish the potential preventative role of topical rifampicin in reducing the occurrence of DSWI.

## Introduction

Although deep sternal wound infections (DSWI) are infrequent after coronary artery bypass grafting (CABG) they still imply significant morbidity and mortality and increased resource utilization^1^. The risk of developing DSWI is dependent on both patients’ co-morbidity profile and procedure related risk factors. Several preventative measures have been identified^2,3^. These include decontamination (nasal and skin), ad hoc antibiotic prophylaxis, meticulous surgical techniques and tight glycemic control. A burgeoning evidence has disclosed that intrawound antibiotics application might significantly reduce the burden of surgical site infection^2–4^. Indeed, despite adequate perioperative antibiotic prophylaxis there might be still an insufficient coverage of common pathogens because diabetes and atherosclerosis impair tissue perfusion. The main advantages of topical antibiotic administration include the high drug concentration and long duration of exposure combined with reduced systemic exposure and, thus, fewer adverse effects^4–7^. On these premises, the present study aimed to determine whether the introduction of topical rifampicin administration might lower the incidence of DSWI. Indeed, despite favorable mechanism of action and pharmacokinetic and proven efficacy on many gram positive and intracellular bacteria, little is known on its usage in the setting of cardiac surgery DSWI^8,9^.

## Materials and methods

### Setting, patient sample and principles of surgical and clinical care

Study setting was the Division of Cardiac Surgery of the “Casa di Cura Montevergine”. This is a private facility in Mercogliano, Avellino, Italy, where nearly 700 patients undergo cardiac surgery annually. Standardized case report forms allow daily collection of clinical parameters from these patients. All of these peri-operative data are entered into an electronic database. Study sample included 965 consecutive patients undergoing CABG via full sternotomy operated on between January 2015 and December 2018. Study design was an observational cohort analysis with 2 sequential patient groups. The control group included those patients operated on between January 2015 and December 2016 (n = 448) prior to the closing protocol change. Those operated on between January 2017 and December 2018 (n = 517) constituted the rifampicin group. In this group, mediastinum, sternum and suprasternal tissues were irrigated after surgery using 600 mg antibiotic powder diluted with 10 ml isotonic solution just before sternal closure. Excluding topical antibiotic administration, several other DSWI preventative measure were standard of care in both cohorts. These included: nasal decontamination, skin antiseptic preparation, prophylactic antibiotic therapy, control of hyperglycemia, parsimonious usage of bone wax, usage of a wide blades sternal spreader, skeletonized internal mammary artery graft harvesting, sternal closure using interlocking figure-of-8 wires and subcutaneous application of povidone-iodine solution before skin closure. Such preventative strategies are in good agreement with recent guidelines and expert consensus documents^2,3^. The protocol for control of hyperglycemia was in accordance with that proposed by the Society of Thoracic Surgeons and did not changed during the study period^10^. As far as the conduits of choice were considered, only skeletonized internal mammary arteries (either isolated left or both arteries) and saphenous vein grafts were harvested. More details on the surgical strategy and postoperative care have been previously reported^11,12^.

### Study aims and clinical outcomes

All definitions were established as part of the original study design. Primary outcome of this study was the incidence of DWSI. The Centers for Disease Control and Prevention classification of surgical site infections^13^ was adopted. Combined results of bacterial cultures, clinical parameters and surgical findings allowed the diagnosis of DSWI. Baseline characteristics and operative risk were defined according to the European System for Cardiac Operative Risk Evaluation (EuroSCORE) II definition criteria^14^. Secondary outcomes were hospital and Intensive Care Unit (ICU) length of stay and all-cause mortality. Study protocol complies with the Helsinki Declaration (as revised in 2013) and was approved by the Clinica Montevergine Ethics and Research Committee (protocol number 29/2017). Since the novel closure protocol was introduced as a standard of care, the need for informed consent was waived.

### Statistical analysis

SAS version 9.4 (SAS Institute, Cary, NC) was employed for all statistical analyses. Continuous variables are reported as mean and SD, whereas categorical variables as absolute number and percentage. Preoperative, operative and postoperative data were compared with unpaired T-test, chi-square and Kruskall-Wallis test. Statistical significance was set at an alpha level of 0.05. To reduce selection bias, a non-parsimonious propensity score was calculated to select two groups of patients with similar baseline characteristics, with patients treated with the new protocol as the fixed group. Those variables known to be associated with a high risk for DSWI were included in the model: age, gender, diabetes, COPD, obesity, extracardiac vascular disease, LVEF, chronic kidney disease, previous cardiac surgery, surgical priority, operative technique, bilateral internal mammary artery usage, perioperative transfusion, any postoperative reoperation^1^. One-to-one propensity score matching was performed using the greedy algorithm and a caliper of 0.2 of the standard deviation of the logit of the propensity score^15^. To evaluate the balance between the matched groups, paired sample t-test for continuous variables, the McNemar test for dichotomous variables, and analysis of the standardized differences after matching have been used. Standardized differences lower than 0.10 were considered an acceptable imbalance between the treatment groups^16^. These tests were used to evaluate any difference in the adverse events of propensity score matched pairs.

## Results

Table 1 summarizes patient population features, surgical priority and preoperative risk scoring. In the unmatched cohort, patients operated during 2015–2016 were more hypertensive and had a greater estimated surgical risk, according to EuroSCORE II. However, propensity matched cohorts proved to be homogeneous.

**Table 1:** Baseline characteristics of the study cohort.

Characteristics	Overall Series				Propensity Score Matched Pairs			
	2015–2016 (n = 448)	2017–2018 (n = 517)	p	Standardized differences	2015–2016 (n = 362)	2017–2018 (n = 362)	p	Standardized differences
Age	66.8±8.9	66.1±8.7	0.16	0.09	66.7±8.7	66.1±8.7	0.34	0.10
Female	89 (19.9)	90 (17.4)	0.33	0.06	63 (17.4)	59 (16.3)	0.76	0.03
Obesity	44 (9.8)	57 (11.0)	0.54	0.04	39 (10.8)	43 (11.9)	0.72	0.04
Systemic hypertension	367 (81.9)	381 (73.7)	0.0023	0.20	287 (79.3)	277 (76.5)	0.30	0.03
Diabetes mellitus	189 (42.2)	216 (41.8)	0.90	0.008	149 (41.2)	153 (42.3)	0.81	0.02
NIDDM	154 (34.4)	182 (35.2)	0.79	0.02	124 (34.3)	121 (33.4)	0.87	0.02
IDDM	35 (7.8)	34 (6.6)	0.46	0.05	25 (6.9)	32 (8.8)	0.38	0.07
Chronic kidney disease	28 (6.3)	39 (7.5)	0.43	0.05	24 (6.6)	30 (8.3)	0.46	0.07
COPD	75 (16.7)	74 (14.3)	0.30	0.07	59 (16.3)	73 (20.2)	0.22	0.10
Extracardiac arteriopathy	49 (10.9)	38 (7.4)	0.05	0.12	32 (8.8)	25 (6.9)	0.37	0.07
LVEF			0.60	0.04			0.054	0.04
>50%	307 (68.5)	367 (71.0)			250 (69.1)	213 (58.8)		
31–50%	121 (27.0)	123 (23.8)			96 (26.5)	122 (33.7)		
21–30%	17 (3.8)	21 (4.1)			13 (3.6)	21 (5.8)		
<21%	3 (0.7)	6 (1.2)			3 (0.8)	6 (1.7)		
Prior cardiac surgery	8 (1.8)	3 (0.6)	0.08	0.11	4 (1.1)	3 (0.8)	>0.99	0.03
Surgical priority			0.82	0.08			0.35	0.08
Elective	412 (92.0)	467 (90.3)			331 (91.4)	312 (86.2)		
Urgent	30 (6.7)	43 (8.3)			28 (7.7)	43 (11.9)		
Emergency	5 (1.1)	6 (1.2)			2 (0.6)	6 (1.7)		
Salvage	1 (0.2)	1 (0.2)			1 (0.3)	1 (0.3)		
EuroSCORE II	2.98±4.73	2.24±3.94	0.0093	0.17	2.91±4.95	2.53±4.62	0.28	0.09

(N)IDDM: (non) insulin-dependent diabetes mellitus; COPD: chronic obstructive pulmonary disease; LVEF: left ventricular ejection fraction.

Operative details are reported in Table 2. Briefly, no differences were observed between groups in terms of number of target vessels, number of grafts, type of graft selected, as well as length of cardiopulmonary bypass, aortic cross-clamping time and operation time.

**Table 2:** Operative details.

Details	Overall Series			Propensity Score Matched Pairs		
	2015–2016 (n = 448)	2017–2018 (n = 517)	p	2015–2016 (n = 362)	2017–2018 (n = 362)	p
Operative technique			<0.0001			0.59
On pump	281 (62.7)	435 (84.1)		276 (76.2)	280 (77.4)	
OPCABG	167 (37.3)	82 (15.9)		86 (23.8)	82 (22.7)	
LIMA	446 (99.6)	513 (99.2)	0.52	359 (99.2)	357 (98.6)	0.48
BIMA	38 (8.5)	31 (6.0)	0.14	28 (7.7)	30 (8.3)	0.89
SVG	382 (85.3)	446 (86.3)	0.67	320 (88.4)	324 (89.5)	0.64
Number of grafts	2.73 ± 0.92	2.84 ± 0.94	0.07	2.68 ± 0.86	2.75 ± 0.87	0.28
CPB (minutes)	86 ± 32	88 ± 37	0.37	86 ± 24	87 ± 35	0.65
Operation time (minutes)	232 ± 57	237 ± 65	0.21	234 ± 58	239 ± 62	0.27

OPCABG: off-pump coronary artery bypass graft; LIMA: left internal mammary artery; BIMA: bilateral internal mammary arteries; SVG; saphenous vein graft; CPB: cardiopulmonary bypass; XCT: aortic cross-clamping time.

Table 3 reports major outcomes in the overall populations as well as in matched cohorts. Deep sternal wound infections were present in 12 patients (1.24%). DSWI were diagnosed early (<30 days) after surgery in most of the cases (11 pts). No relation was found between incidence of DSWI and surgical technique (OPCABG OR 0.342, 95% CI 0.192–1.71, p = 0.07). Similarly, number of grafts (OR 1.213, 95% CI 0.397–2.341, p = 0.12) and surgical time (OR 2.851, 95% CI 0.875–4.382, p = 0.23) did not emerged as univariate predictors of DSWI.

**Table 3:** Postoperative outcomes.

Details	Overall Series			Propensity Score Matched Pairs		
	2015–2016 (n = 448)	2017–2018 (n = 517)	p	2015–2016 (n = 362)	2017–2018 (n = 362)	p
In-hospital stay (days)	13.8 ± 8.6	12.8 ± 6.0	0.054	13.3 ± 8.3	13.3±8.7	>0.99
ICU stay (days)	2.4 ± 3.0	2.1 ± 2.2	0.07	2.3 ± 2.3	2.3±2.5	0.98
Reoperation	18 (4.0)	6 (1.2)	0.0045	5 (1.4)	6 (1.7)	>0.99
Bleeding	15 (3.3)	6 (1.2)		4 (1.1)	6 (1.7)	
Graft failure	3 (0.7)	0		1 (0.3)	0	
RBC transfusion	194 (43.3)	202 (39.1)	0.18	145 (40.1)	152 (42.0)	0.65
DSWI	11 (2.5)	1 (0.2)	0.0016	8 (2.1)	1 (0.3)	0.0391
SVG wound infection	3 (0.67)	3 (0.58)	0.86	2 (0.55)	1 (0.28)	0.56
Death	8 (1.8)	6 (1.2)	0.42	5 (1.4)	6 (1.7)	>0.99

ICU: intensive care unit; DSWI: deep sternal wound infection; SVG: saphenous vein graft.

Patients in the rifampicin group performed significantly better (1 vs 11 DSWI, 2.5% vs 0.2%). The percentage of Gram-positive infections was 100%. Positive swabs for Coagulase Negative Staphylococcus species accounted for 75% of the cases. There were 2 cases of staphylococcus aureus infections (one in each study period, with no methicillin resistant strain) and 1 case of enterococcus faecalis isolation. Negative pressure wound therapy was applied in all cases. Final closure technique was sternal re-wiring in 4, pectoral muscle flap in 6 and subcutaneous closure in 2 patients. No adverse reaction has been observed in the group of patients receiving topical rifampicin. Incidence of SVG wound infections was similar in both groups, but topical antibiotic application was limited to the chest incision.

As to secondary outcomes, no significant difference emerged for hospital and ICU length of stay. Similarly, no difference emerged as to hospital mortality. Noteworthy, all patients experiencing DSWI were discharged home or to a rehabilitation program, according their clinical conditions.

## Discussion

DSWI significantly jeopardise hard outcomes and impact on the overal periprocedural economic burden^17,18^. This study shows that topical rifampicin in combination with commonly prescribed preventive strategies significantly reduces the incidence of DSWI in an unselected cohort of CABG patients operated on through a full median sternotomy. The overall incidence of DSWI was 1.24%, a rate that closely matches what has been previously reported^11,19^. This rate was reduced to a promising 0.2% by the new closing protocol. As reported in a recent authoritative literature review, given several biases, the variation in the incidence of DSWI is so high to preclude any systematic analysis. In other words it is still impossible to define a benchmark standard for outcome comparison^20^. A recent Japanese nationwide survey on cardiac surgical procedures, demonstrated that CABG surgery, despite implying the lowest odds ratio for operative mortality, actually entails the highest potential for DSWI development (1.36 [95% CI: 1.24–1.49]) and 1.52 [95% CI: 1.32–1.76] respectively)^21^. In the same analysis it is also shown that DSWI should be considered independently of the surgical mortality. Indeed those hospital reachig the lowest risk-adjusted mortality rate do not always have the same performance as to the incidence of DSWI^17^. This tertiary care center displays an high case load (>220 cases per year) with a satisfactory mortality rate (overall 1,4%). Topical application of antibiotics has been recently recommended by expert consensus statements^2,3^ despite an unpredictable potential for the developent of antibiotic resistance. Topical vancomycin and gentamicin have been recommended as a standard of care even though the level of evidence is low and published data are somewhat conflicting^2,3,5,6^. The burden of bacterial biofilms is highest in musculoskeletal infections as DSWI. Vancomycin has minimal eradication effect on this respect. *In vitro* and *in vivo* studies have shown that Rifampicin is an effective Staphylococcal biofilm eradicator with no adverse effects on bone healing^7^. Rifampicin’s mechanism of action is independent of cellular replication since its target is bacterial protein synthesis. This antibiotic displays strong bactericidal effect on both gram positive and negative species. Other relevant features include: unexpensiveness and easiness of preparation and and handling. It has also been consistently shown to decrease wound infection in other surgical settings^4,7,22^. The present study is the first large scale trial to evaluate its preventive potential. It expands the findings of the pivotal study by Aygun and colleagues with the inherent merit of an all comer design which is more close to a real world setting. More, it enclosed procedure performed both on pump and off-pump as well as a fair number of procedures with bilateral mammary artery harvesting which are representative of current surgical practice pattern^23^. Several methodological features of the present analysis should be underscored for a thourough data evaluation. Major strengths of this study are: the adherence to standardised definition of both patients feauters and outcomes events, and the single centre setting along with its inherent standardised perioperative care pathway. Such a combination significantly limited the variability due to the influence of individual and institutional practice on hospital and ICU length of stay and pattern of resource utilisation. A benchmark for outcomes measures in morbidity studies. Specularly, the single center setting might imply one-sided results not readily transferable to other patient populations due to the influence of specific standards of clinical practice and a unique patient population features. More, it is a retropsective analysis of prospectively collected data^24^. Though all known risk factors have been accounted for, there might be still a potential for unknown predictors. Morover, the study involves separate time periods. Nevertheless, the large sample size, no change in perioperative management algorithms, and accurate propensity matching for predictors of DSWI should have prevented major biases. Finally, despite the large study sample, the study analysis may still be statistically underpowered. Indeed, the relatively small number of index cases, which prevented any meaningful regression analysis for DSWI development, might theroretically imply a definite probability of type II error.

## Conclusions

Topical rifampicin in combination with known preventive perioperative strategies significantly reduces the incidence of DSWI after CABG. Clearly, our study is hypothesis-generating given its proper nature, and to finally clarify the issue at stake, further trials are mandatory.
